# Folate Production by Probiotic Bacteria

**DOI:** 10.3390/nu3010118

**Published:** 2011-01-18

**Authors:** Maddalena Rossi, Alberto Amaretti, Stefano Raimondi

**Affiliations:** Department of Chemistry, University of Modena and Reggio Emilia, via Campi 183, Modena 41100, Italy; Email: alberto.amaretti@unimore.it (A.A.); stefano.raimondi@unimore.it (S.R.)

**Keywords:** folate, probiotic, *Lactobacillus*, *Bifidobacterium*, microbiota, gut

## Abstract

Probiotic bacteria, mostly belonging to the genera *Lactobacillus* and *Bifidobacterium*, confer a number of health benefits to the host, including vitamin production. With the aim to produce folate-enriched fermented products and/or develop probiotic supplements that accomplish folate biosynthesis *in vivo* within the colon, bifidobacteria and lactobacilli have been extensively studied for their capability to produce this vitamin. On the basis of physiological studies and genome analysis, wild-type lactobacilli cannot synthesize folate, generally require it for growth, and provide a negative contribution to folate levels in fermented dairy products. *Lactobacillus plantarum* constitutes an exception among lactobacilli, since it is capable of folate production in presence of para-aminobenzoic acid (pABA) and deserves to be used in animal trials to validate its ability to produce the vitamin *in vivo*. On the other hand, several folate-producing strains have been selected within the genus *Bifidobacterium*, with a great variability in the extent of vitamin released in the medium. Most of them belong to the species *B. adolescentis* and *B. pseudocatenulatum*, but few folate producing strains are found in the other species as well. Rats fed a probiotic formulation of folate-producing bifidobacteria exhibited increased plasma folate level, confirming that the vitamin is produced *in vivo* and absorbed. In a human trial, the same supplement raised folate concentration in feces. The use of folate-producing probiotic strains can be regarded as a new perspective in the specific use of probiotics. They could more efficiently confer protection against inflammation and cancer, both exerting the beneficial effects of probiotics and preventing the folate deficiency that is associated with premalignant changes in the colonic epithelia.

## 1. Introduction

The consumption of live microbial supplements with presumptive health benefits on human physiology, the so-called probiotics, has become a common practice. Probiotic bacteria positively impact on the immune system and on the composition and functioning of the gut microbiota. Furthermore, the production of vitamins has been claimed among the causal relationships of the healthy benefits of probiotics. Folates represent an essential nutrition component in the human diet, being involved in many metabolic pathways. The daily recommended intake as approved in the European Union is 400 μg/day for adults [1,2]. Efficiency of DNA replication, repair and methylation are affected by folate, therefore high amounts of folate are required by fast proliferating cells such as leucocytes, erythrocytes and enterocytes [3]. Epidemiological studies indicated that folate deficiency is often associated with increased risk of breast cancer and that low folate homeostasis may induce hypomethylation of DNA, thereby promoting cancer on the proliferating cells of the colorectal mucosa that supports rapid and continuous renewal of the epithelium [4,5]. Furthermore, increased folate intake is recommended also for patients with inflammatory bowel diseases, contributing to regulation of rectal cell turnover [6]. 

## 2. The Intestinal Microbiota and Vitamins

The human colon harbors a complex and dense microbial population, with up to 10^11^ microorganisms per gram of intestinal content, mostly represented by anaerobic bacteria. This microbiota also includes archaea, yeasts, and other eukaryotes. Although more than 50 bacterial phyla have been described, it is dominated by *Firmicutes*, *Bacteroidetes*, *Actinobacteria*, and *Proteobacteria* [7]. Within these phyla, the number of different bacterial species and strains is extremely high, accounting for several thousands of diverse microorganisms.

The gut microbiota benefits the host, playing a pivotal role in nutrient digestion and energy recovery. Colonic bacteria produce enzymes that the host lacks, which are involved in breakdown of complex molecules, such as plant polysaccharides. The fermentation of the dietary components that escape digestion and absorption in the upper intestinal tract, and of endogenous products such as mucin, results in production of organic acids (e.g., acetic, lactic, propionic, and butyric acids), branched chain fatty acids (e.g., isobutyric, isovaleric, and 2-methylbutyric acids), H_2_, CO_2_, ammonia, amines and several other end-products. These fermentation products affect the gut environment and the host health, acting as energy sources, regulators of gene expression and cell differentiation, and anti‑inflammatory agents. In fact, host-microbe interactions are essential for the resistance to pathogenic infections, gut development, and epithelial homeostasis [8,9]. 

The gut microbiota has also been recognized as a source of vitamins. They cannot be synthesized by mammals and must be obtained via intestinal absorption from exogenous sources, such as the diet and the gut microbiota. Germ-free animals need to be supplemented with vitamin K and certain B vitamins. In conventionally colonized animals, these vitamins are produced by several intestinal genera, for instance *Bacteroides* and *Eubacterium*. Furthermore, it is well established that the ruminal microbiota is a rich source of vitamins to the ruminant and that the fecal bacteria are a major source for coprophagic rodents [10]. Evidence that commensal colonic bacteria are a significant source of a range of vitamins to humans has been presented as well [11].

The microbiota of the human colon is known to produce vitamin K (menaquinones) and most of the water-soluble vitamins of group B, including biotin, nicotinic acid, folates, riboflavin, thiamine, pyridoxine, panthotenic acid, and cobalamin [11]. In fact, the whole genetic information of the microbial community (microbiome) of the human distal gut revealed a variety of COGs (Clustered Orthologous Groups) which are involved in the synthesis of several essential vitamins [12]. Unlike dietary vitamins, which are mainly absorbed in the proximal part of the small intestine, the uptake of microbial vitamins predominantly occurs in the colon [13]. Colonocytes appear to be able to absorb biotin, thiamin, folates, riboflavin, panthotenic acid, and menaquinones, indicating that the microbiota-produced vitamins may contribute to the systemic vitamin levels and especially to the homeostasis of the vitamins in the localized epithelial cells [13,14]. 

Folates are hydrophilic anionic molecules that do not cross biological membranes by diffusion, but specialized membrane transport systems allow folate accumulation into mammalian cells and tissues. Absorption exploits several genetically and functionally distinct transporters, such as the folate receptors, the family of organic anion transporters, a proton-coupled folate transporter, and the reduced folate carrier, which is ubiquitously expressed [15]. Each mechanism plays a unique role in mediating the transport across epithelia and into systemic tissues, and contributes to folate homeostasis in humans [16]. Even though absorption occurs primarily in the duodenum and upper jejunum, the colon represents a major depot of folate and the vitamin produced by the colonic bacteria exceeds dietary intake and affects the folate status of the host. It is produced in large quantities by the colonic microbiota, mainly as monoglutamylated folate, the form that is absorbed at the highest rate [17], intestinal bacteria being one source of this vitamin. Many studies assessed the contribution of intestinal microbiota to the folate intake of animal hosts [17,18,19,20], and it has been demonstrated that the folate synthesized by intestinal bacteria can be absorbed and used by the host [18,21,22,23,24]. Recently, direct evidence of absorption of folate across the colon has been irrefutably provided [25]. The apparent rate of absorption in the colon is considerably lower than that in the small intestine. However, in the distal portion of the gastrointestinal tract the transit time is longer than in the small intestine, and the supply of folates by the colonic microbiota is constant and continuous, whereas their availability in the upper tract is discontinuous and mostly affected by food intake.

## 3. Probiotics

Disturbance of the proper balance of intestinal microbiota is involved in several pathologies, such as inflammatory bowel diseases, metabolic diseases, cancer, and autoimmune diseases. Besides, specific intestinal bacteria have been claimed as therapeutic or prophylactic against infections and several diseases, and are used as probiotics [26]. Probiotics are defined as “live microbes which, when administered in adequate amounts, confer a health benefit to the host” [27]. Another definition recognizes probiotic as “a preparation of or a product containing viable, defined microorganisms in sufficient numbers, which alter the microflora (by implantation or colonization) in a compartment of the host and by that exert beneficial health effects to this host” [28]. These nonpathogenic organisms do not necessarily possess a phylogenetic relation to one another and are best defined functionally rather than taxonomically. Probiotic microorganisms have been identified within different phyla of bacteria and yeasts, since a variety of strains of bifidobacteria, lactobacilli, enterococci, streptococci, propionibacteria, *Bacillus* sp., *Escherichia coli*, and yeasts have been claimed to exert beneficial activities on human health [29]. However, the majority of probiotics in use today belong to the genera *Lactobacillus* and *Bifidobacterium*. 

The function of the probiotic bacteria, which can be added to foods or consumed as pharmaceutical products, includes the reduction of potential pathogenic bacteria and/or harmful metabolites in the intestine and the normalization of gastrointestinal functions modulating immunological parameters, intestinal permeability and bacterial translocation, or providing bioactive or otherwise regulatory metabolites. Most health effects of probiotic microorganisms are determined by interactions with immunocompetent cells of the intestinal mucosa. Indeed, the gut-associated lymphoid system is the largest immunologically competent organ in the body, and maturation and optimal development of the immune system since birth depends on the development and composition of the indigenous microbiota [29]. While at first, probiotics were consumed to modulate and improve the gut microbiota balance, nowadays specific health effects have been established, and they are supplied to alleviate chronic intestinal inflammatory diseases, to prevent and treat pathogen-induced diarrhea, or to manage autoimmune and atopic diseases [26,30,31,32].

## 4. Biosynthesis of Folate

Both prokaryotic and eukaryotic cells require reduced folate cofactors as acceptor/donor of one-carbon units in a variety of biosynthetic processes, including the formation of methionine, purines, and thymine, and in some degradative reactions. While the cellular requirement for folates is universal, methods for obtaining them differ among organisms. Animals cannot synthesize folates and assimilate these derivatives with a diet exploiting active transport systems. Diversely, plants, fungi, certain protozoa, and several archaea and bacteria can synthesize folates *de novo*, likely through the same general biosynthetic pathway [33] with some modifications [34,35,36,37,38].

**Figure 1:**
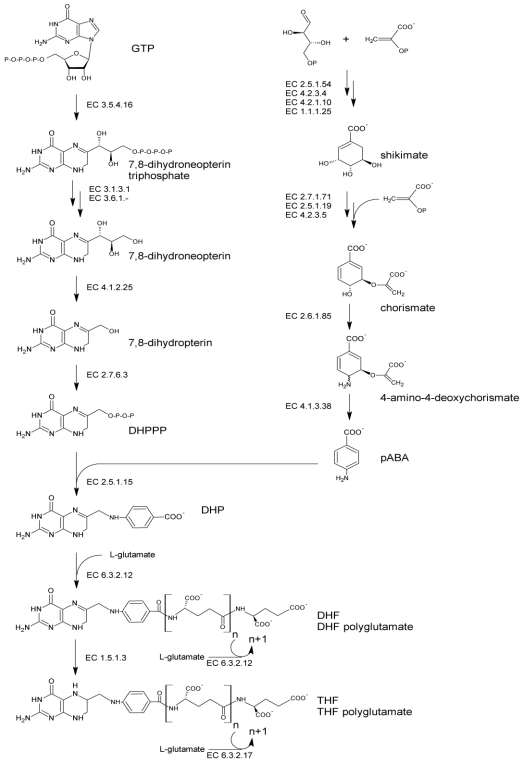
Pathway of *de novo* bacterial biosynthesis of folate. Abbreviations: GTP, guanosine triphosphate; DHPPP, 6-hydroxymethyl-7,8-dihydropterin pyrophosphate; pABA, para-aminobenzoic acid; DHP, 7,8-dihydropteroate; DHF, dihydrofolate; THF, tetrahydrofolate.

The folate molecule contain one pterin moiety, originating from 6-hydroxymethyl-7,8-dihydropterin pyrophosphate (DHPPP), bound to para-aminobenzoic acid (pABA, vitamin B10). Thus, *de novo* biosynthesis (Figure 1) necessitates both the precursors, DHPPP and pABA. The latter can be produced by plants and bacteria from the pentose phosphate pathway. Erythrose 4-phosphate and phosphoenolpyruvate undergo the shikimate pathway to ultimately lead to chorismate, which serves as a branching point toward the biosynthesis of aromatic amino acids and pABA. Chorismate is converted via aminodeoxychorismate synthase (EC 2.6.1.85) into 4-amino-4-deoxychorismate. Subsequently, pyruvate is cleaved by 4-amino-4-deoxychorismate lyase (EC 4.1.3.38) to give pABA, which ultimately serves for folate biosynthesis. The biosynthesis of DHPPP proceeds via the conversion of guanosine triphosphate (GTP) in four consecutive steps. The first step is catalyzed by GTP cyclohydrolase I (EC 3.5.4.16) and involves an extensive transformation of GTP, through Amadori rearrangement, to form a pterin ring structure. Following dephosphorylation, the pterin molecule undergoes aldolase and pyrophosphokinase reactions, which produce the activated pyrophosphorylated DHPPP. 

Folate biosynthesis continues with the formation of a C–N bond joining DHPPP to pABA. This condensation reaction, catalyzed by dihydropteroate synthase (EC 2.5.1.15), yields 7,8-dihydropteroate (DHP). DHP is glutamylated by dihydrofolate synthase (EC 6.3.2.12) giving dihydrofolate (DHF). Then, it is reduced by DHF reductase (EC 1.5.1.3) to the biologically active cofactor tetrahydrofolate (THF) and subjected to the addition of multiple glutamate moieties by folylpolyglutamate synthase (EC 6.3.2.17) to yield THF-polyglutamate. Polyglutamilation may take place also before the occurrence of the reduction step, being catalyzed by DHF synthase or, in many bacteria, by a bifunctional enzyme which is responsible for both EC 6.3.2.12 and EC 6.3.2.17 activities [38].

## 5. Production of Folate by Lactobacilli

The genus *Lactobacillus* includes almost two hundreds recognized species of low G + C gram‑positive eubacteria within the phylum of the *Firmicutes* and the *Clostridium-Bacillus* subdivision [39]. Despite their wide phylogenetic and functional diversity, lactobacilli are invariably anaerobic/microaerophilic, aciduric/acidophilic non-sporulating rods. Lactobacilli are included within the functional group of lactic acid bacteria (LAB), being saccharolytic and strictly gaining energy through the lactic fermentation of carbohydrates, and can be classified as obligate homo-fermentative (giving mainly lactic acid), obligate hetero-fermentative (giving mainly lactic acid, acetic acid, and CO_2_), or facultative hetero-fermentative [40].

Lactobacilli occur in a variety of habitats, including plant-derived matrices, fermented foods (such as dairy products and fermented dough, milk, vegetables, and meats), and diverse niches within the body of humans and animals. In particular, several species are endogenous members of the resident microbiota of the hindgut. Many commensal lactobacilli have been proven to exert a number of beneficial health effects and have attracted considerable attention as probiotics, although the molecular mechanisms behind these beneficial properties are still under investigation. Besides, lactobacilli of food origin are primarily important in the production of fermented products, but are increasingly investigated for the production of healthy functional foods. At present, the strains of *Lactobacillus* with the greatest relevance for the manufacturing of probiotics and functional foods belong to the species *L. acidophilus*, *L. casei*, *L. paracasei*, *L. plantarum*, *L. reuteri*, and *L. salivarius* [26,29].

Due to potentially relevant applications, the ability to produce folate has been intensively investigated in many *Lactobacillus* isolates from a variety of origins. Strains from the human gastrointestinal tract could find application as folate-producing probiotics, while strains from fermented foods could be exploited as microbial starters for manufacturing folate-fortified dairy products with improved nutritional value. In this perspective, efforts were accomplished to investigate the vitamin requirements of lactobacilli and to determine the effects of their growth on folate levels in diverse media [41,42,43,44,45]. Recently, the genome sequence of an increasing number of strains of *Lactobacillus* and LAB has provided a major contribution to the knowledge of folate biosynthesis by these bacteria [38], even if the number of genomes sequenced is still limited, compared to the total amount of species.

The analysis of genome sequences for predictable metabolic pathways using KEGG database [46] suggests that the ability to synthesize pABA *de novo* is absent among all the sequenced members of the genus *Lactobacillus* (Table 1). In fact, the enzymes which are necessary for chorismate conversion into pABA are lacking. Moreover, the shikimate pathway for chorismate production is complete only in the strains of *L. plantarum*, while it is absent or partial in all the other lactobacilli. Thus, it is expected that lactobacilli are generally unable to produce folate in the absence of pABA. Diversely, pABA supplementation should be unnecessary in the phylogenetically related genera *Lactococcus* and *Streptococcus* [40], since all the sequenced lactococci and streptococci, with rare exceptions, possess all the genes for both shikimate pathway and chorismate conversion into pABA.

**Table 1:** Genes and enzymes for the biosynthesis of DHPPP, THF-polyglutamate, chorismate, and pABA predicted from the sequenced genomes of genus *Lactobacillus* and other lactic acid bacteria [46]. Abbreviations: pABA, para-aminobenzoic acid; DHPPP, 6-hydroxymethyl-7,8-dihydropterin pyrophosphate; THF, tetrahydrofolate.

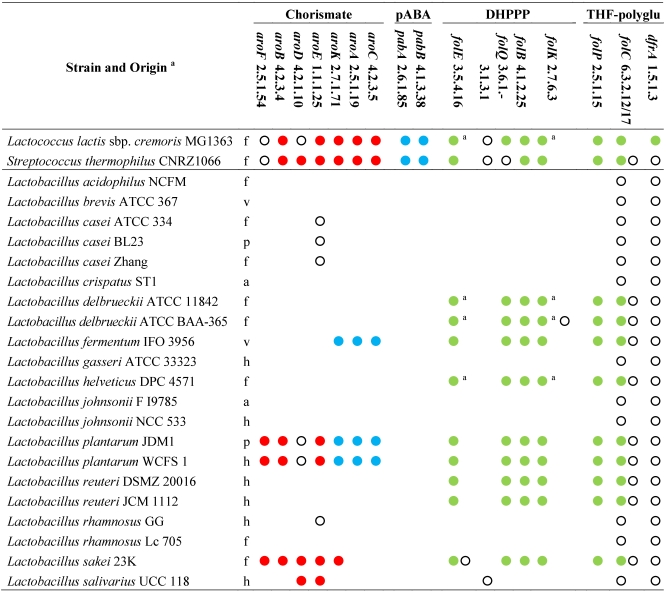

^a^, h: human gastro-intestinal tract; a: animal gastrointestinal tract; p: probiotic; f: fermented food (meat or dairy products); v: plant; Within each strain, dots with the same color indicate genes organized within the same gene cluster; empty dots indicate genes located elsewhere in the genome; Within each strain, dots with the same letter indicate the same gene encoding different enzymatic activities.

The sequenced strains of *L. johnsonii*, *L. acidophilus*, *L. salivarius*, *L. brevis*, *L. casei*, *L. gasseri*, *L. rhamnosus*, and *L. crispatus* lack the genes of DHPPP *de novo* biosynthetic pathway and also the gene encoding dihydropteroate synthase (EC 2.5.1.15), whereas they possess the genes for DHP transformation into DHF, THF, and THF-polyglutamate. Therefore, it is expected that these strains are auxotrophic for folates or DHP, and remain incapable of folate production even in the presence of pABA supplementation.

Like *Lactococcus lactis*, the sequenced strains *L. plantarum*, *L. sakei*, *L. delbrueckii*, *L. reuteri*, *L. helveticus*, and *L. fermentum* harbor a folate biosynthesis cluster that includes the gene encoding dihydropteroate synthase (EC 2.5.1.15) and all the genes for the biosynthesis of DHPPP, with the exception of alkaline phosphatase (EC 3.1.3.1). In *L. lactis*, the dephosphorylation of dihydroneopterin triphosphate into the monophosphate has been demonstrated to occur through an alternative route and to involve a Nudix pyrophosphohydrolase (with provisional number EC 3.6.1.-) [47]. Most of lactobacilli harbor a number of genes encoding putative Nudix enzymes (including *mutT* genes for DNA repair), but only *L. sakei*, *L. helveticus*, and *L. delbrueckii* have a homolog of *L. lactis* gene within the *fol* cluster. Diversely, in *L. fermentum*, *L. plantarum*, and *L. reuteri*, the *fol* cluster contains the gene of a putative non-Nudix purine NTP pyrophosphatase, which is probably responsible for hydrolyzing dihydroneopterin triphosphate in these species. Therefore, *L. plantarum*, *L. sakei*, *L. delbrueckii*, *L. reuteri*, *L. helveticus*, and *L. fermentum* are expected to produce DHPPP and may be considered as potential folate producers if they are cultured in the presence of pABA.

Many authors have investigated the ability of LAB to grow in folate-free media and to produce folate. *L. casei* was among the first folate-auxotrophic bacteria to be discovered [48] and among the first organisms whose folate uptake system was biochemically described [49,50,51]. In *L. casei* and *L. salivarius*, the latter being another folate-auxotrophic species, the uptake proceeds via an abundant high affinity membrane-associated binding protein which facilitates the passage of folate across the membrane as an electroneutral complex with cations, with an influx that is half-maximal at folate concentrations in the nanomolar range. The uptake system of *L. casei* has been cloned and characterized, and has been classified within the new class of prokaryotic transporters based on a shared energy-coupling factor (ECF). It is based on an ECF component, shared with the transport systems of thiamine and biotin, plus a folate-specific binding protein. The specific component is encoded by the gene *folT*, which has homologs in most of lactobacilli and in other *Firmicutes*, where the corresponding genes may be included within the *fol* cluster [52,53].

Within the cell, the substrate slowly dissociates from internalized binding sites and is sequentially metabolized to coenzyme forms and then to membrane-impermeable folylpolyglutamates [49,54,55]. More recently, dozens of strains of LAB have been screened for folate production. Unlike the strains of *Lactococcus lactis* subsp. *cremoris*, *Lactococcus lactis* subsp. *lactis*, and *Streptococcus thermophilus*, the strains of *Lactobacillus* are generally unable to produce folate with the exception of *L. plantarum* [42,56]. The strains of *Lactococcus lactis* and *Streptococcus thermophilus* were demonstrated to produce folate, to accumulate the vitamin within the cells, and excrete it into the medium. The extent of vitamin production, the partitioning between accumulation and excretion, and the form in which the vitamin occurred (e.g., the number of glutamate residues, and the association to formyl or methenyl groups) mostly depended on the strain and, in some cases, were influenced by culture conditions, such as the pH, the growth rate and the presence of pABA. On the contrary, the strains of *Lactobacillus* consumed folate with the exception of *L. plantarum*. With hindsight, these observations are in agreement with the presence or the lack of the genes for folate biosynthesis, as predicted from the sequenced genomes.

Several attempts were carried out to exploit strains of *Lactobacillus* for folate fortification of fermented dairy products, but the use of just lactobacilli is likely to deplete the folate levels of the fermented product [56,57,58,59,60,61]. Nonetheless, folate production and utilization is additive in mixed cultures of *S. thermophilus* and lactobacilli. Thus, increased folate levels in yoghurt and fermented milk are possible through judicious selection of inoculum species, even though the folate levels remain relatively low in terms of recommended daily intake [56].

Combining genome-based metabolic models with growth experiments on minimal media is fundamental to unravel the authentic metabolic capabilities and nutritional requirements of bacteria and to reveal inconsistencies between the predictions and their actual behavior. Specifically, it was found that amino acids, bases and nucleosides present in non-minimal media could circumvent the need for specific cofactors such as folate and pABA [62,63]. Genome-based predictions and the utilization of chemically defined media have been satisfactorily combined only for *L. plantarum* so far [45]. Using a medium lacking all components needed for folate production or folate-dependent metabolite formation, it was demonstrated that, in the presence of pABA, *L. plantarum* produced a surplus of folate that exceeded the requirement of its own metabolism. The absence of pABA suppressed the production, but did not affect growth rate or biomass formation at all, thus the presence of an alternative cofactor for the one-carbon donation may not be excluded.

*In silico* analysis of the folate biosynthesis genes of the B12 producer *L. reuteri* JCM1112 was used to develop a metabolic engineering strategy and to optimize the composition of pABA-supplemented fruit-based media aiming at combining the production of B12 and folate in the desired ratio [43]. The overexpression in *L. reuteri* of the complete folate biosynthesis gene cluster from *L. plantarum* increased folate production to levels substantially higher than those previously described, even though these recombinant strains cannot be directly used by the food industry.

## 6. Production of Folate by Bifidobacteria

*Bifidobacterium* is a genus of high G + C Gram-positive eubacteria within the phylum of *Actinobacteria*. They are saccharolytic obligate anaerobes whose primary habitat is the gastrointestinal tract of animals, being among the first gut colonizers. Among nearly fifty species recognized so far [39], the most represented in the gastrointestinal tract of human adults or infants, are *Bifidobacterium pseudocatenulatum*, *B. catenulatum*, *B. adolescentis*, *B. longum*, *B. infantis*, *B. breve*, *B. angulatum* and *B. dentium* [64]. Bifidobacteria are one of the most important health-promoting groups of the colonic microbiota and one of the most important microorganisms to be used as probiotics. Several reports provided insight into metabolic, trophic and protective functions that reinforce the functional claims of bifidobacteria. They produce lactic and acetic acids which acidify the large intestine and restrict putrefactive and potentially pathogenic bacteria, inhibit the attachment and the growth of transient organisms and pathogens, repress harmful enzymatic activities within the microbiota, activate a number of dietary compounds into bioactive healthy molecules, and produce vitamins and amino acids. Furthermore, they have been demonstrated to participate in the regulation of intestinal homeostasis, modulate local and systemic immune responses, and play an important role in the protection against cancer and inflammatory diseases [29,65]. However, the mechanisms of action are not yet completely understood and it is likely that more than one mechanism works simultaneously to bring about the health benefits [65].

Among intestinal bacteria, bifidobacteria are generally considered to synthesize several B group vitamins, including folate, biotin, thiamine, nicotinic acid, pyridoxine, riboflavin, and B12, but have not been reported to produce vitamin K. Nonetheless, the capability of bifidobacteria to produce vitamins and to release them extracellularly has never been explored in depth, with the sole exception of folate [22,66,67]. In fact, the lack of suitable analytical methods with sufficient sensitivity, and of a general synthetic medium where most *Bifidobacterium* spp. abundantly grow, has delayed an extensive screening of *Bifidobacterium* strains for auxotrophies, synthesis, and secretion of vitamins.

Based on the analysis of genome sequences for predictable metabolic pathways [46], all the sequenced bifidobacteria possess the entire set of the genes for the shikimate pathway and are expected to produce chorismate (Table 2). Even though they harbor the gene encoding the aminodeoxychorismate synthase (EC 2.6.1.85), only the strains of *B. adolescentis* and *B. dentium* possess the 4-amino-4-deoxychorismate lyase (EC 4.1.3.38) and are expected to accomplish *de novo* biosynthesis of pABA. Both the sequenced strains of *B. animalis* subsp. *lactis* lack the gene encoding dihydropteroate synthase (EC 2.5.1.15) and all the genes for the biosynthesis of DHPPP, thus they should behave auxotrophic for folates or DHP, and remain incapable of folate production even in the presence of pABA supplementation. 

**Table 2:** Genes and enzymes for the biosynthesis of DHPPP, THF-polyglutamate, chorismate, and pABA predicted from the sequenced genomes of genus *Bifidobacterium* [46]. Abbreviations: pABA, para-aminobenzoic acid; DHPPP, 6-hydroxymethyl-7,8-dihydropterin pyrophosphate; THF, tetrahydrofolate.

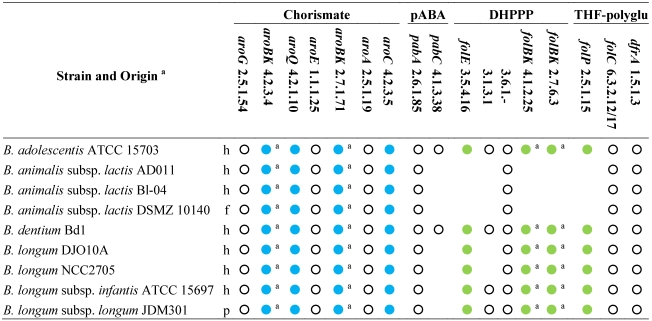

^a^, h: human gastro-intestinal tract; p: probiotic; f: fermented food (meat or dairy products); Within each strain, dots with the same color indicate genes organized within the same gene cluster; empty dots indicate genes located elsewhere; Within each strain, dots with the same letter indicate the same gene encoding different enzymatic activities.

All the other sequenced bifidobacteria harbor a cluster of *fol* genes encoding dihydropteroate synthase (EC 2.5.1.15) and some enzymes for the biosynthesis of DHPPP. Therefore, bifidobacteria are expected to carry out the condensation between pABA and DHPPP. Unlike lactobacilli, the *fol* cluster of bifidobacteria encodes for neither pyrophosphohydrolase (EC 3.6.1.-) nor alkaline phosphatase (EC 3.1.3.1). Several putative Nudix pyrophosphatases and alkaline phosphatase are widespread in all the sequenced genomes of bifidobacteria, but it is not possible to argue whether they are involved in folate biosynthesis, because their function has not been investigated so far. Therefore, it should not be excluded that *B. adolescentis*, *B. dentium*, and *B. longum* can accomplish the dephosphorylation of dihydroneopterin through an enzyme so-far unidentified or an enzyme‑independent chemical process, and can synthesize DHPPP. Furthermore, it is conceivable that *B. adolescentis* and *B. dentium* are capable of *de novo* folate production, while *B. longum* needs to be provided with pABA, and *B. animalis* requires folate.

Several strains of bifidobacteria have been screened for their ability to produce folate in low-folate or folate-free media. Twenty-four strains of *B. bifidum*, *B. infantis*, *B. breve*, *B. longum*, and *B. adolescentis* were cultured in a low-folate semisynthetic medium and significant differences in vitamin accumulation were found among the species tested [66]. All *B. bifidum* and *B. infantis* strains were classified as high folate accumulators, while *B. breve*, *B. longum*, and *B. adolescentis* produced lower amounts of the vitamin. For all the strains, extracellular folate accounted for most of the accumulated vitamin [68]. In other studies, the highest folate accumulation in reconstituted skim-milk was obtained after incubation with *B. breve* and *B. infantis* or *B. longum* strains [69].

Rather different results were obtained when 76 wild-type strains were screened in a folate-free semisynthetic medium [66]. Supplementation of folate was necessary for the growth of most of the strains, and the ability to produce the vitamin in the folate-free medium was found only in 17 strains belonging to nine different species (*B. adolescentis*, *B. breve*, *B. pseudocatenulatum*, *B. animalis*, *B. bifidum*, *B. catenulatum*, *B. dentium*, *B. infantis*, and *B. longum*). The vitamin production was not related to the extent of the growth, and was not a characteristic of the species, but seemed to be a trait of the single strains. The vitamin was mainly extracellular; intracellular accumulation was strain‑dependent and ranged between 9 and 38% of total vitamin production. The highest extracellular folate levels (between 41 and 82 ng mL^−1^) were produced by four strains of *B. adolescentis* and two of *B. pseudocatenulatum*. Only one out of 15 *B. longum* strains grew in folate free-medium.

The discrepancy with the studies that did not identify *B. adolescentis* and *B. pseudocatenulatum* as high-producers may be due to both strain-to-strain differences and to different experimental designs [66,68]. Unlike the previous studies, folate-free medium was used, and the cultures were passaged seven times in this medium to exhaust the vitamin before evaluating growth and folate production [66]. Furthermore, it is conceivable that several strains have been phenotypically identified and classified when the number of species was lower, and need reclassification based on molecular phylogenetic analyses. 

In the perspective to develop a probiotic based on folate-producing strains, it is important that vitamin biosynthesis is not affected by the environmental conditions occurring in the colon, and particularly by the level of exogenous vitamin, whose concentration range can be rather large depending on the dietary intake, absorption and excretion from urine, skin and bile [6]. Among the above strains, two *B. adolescentis* and one *B. pseudocatenulatum* were selected since they did not exhibit any feed-back regulation of folate production, due to the presence of exogenous vitamin in the range between 0 and 50 ng mL^−1^ [66]. Furthermore, neither pH nor the carbon source affected folate biosynthesis. These selected strains were administered to Wistar rats with induced folate deficiency, in order to investigate their effectiveness to improve folate status [22]. Lyophilized bifidobacteria were used alone or were added to bifidogenic fructans in a synbiotic formulation. At the end of the treatment, mean serum folate concentration in rats consuming both the probiotic and synbiotic diet was significantly higher than in controls. However, the simultaneous consumption of probiotics and prebiotic carbohydrates further increased the level of the probiotic strains in the intestine and resulted in the highest level of serum folate, confirming that the availability of a preferred indigestible carbon source is advantageous for the growth and the metabolic activity of probiotic bacteria. 

These same strains of *B. adolescentis* and *B. pseudocatenulatum*, when given to 23 healthy volunteers in a pilot human study, significantly increased folate concentration in the feces of the subjects [67]. These results corroborate the assumption that the increase of folate levels was markedly due to the effective growth of the folate-producing bifidobacteria. In this case, the levels of commensal bifidobacteria in the large intestine correlated with the vitamin availability, suggesting that bifidobacteria are capable of producing folate in the gut, and that the folate synthesized in the large intestine can be absorbed and utilized by the host. In agreement with these results, in folate-depleted rats the administration of diets containing bifidogenic ingredients (e.g., human milk solids or prebiotics) increased the folate concentration in the cecum, colon, plasma, and colonic tissue [70,71]. These results support evidence that folate-producing probiotic strains may represent an endogenous source of vitamin, preventing its deficiency in the colon. Localized folate production in the large intestine may provide the proliferating enterocytes with this essential vitamin with potential effects in reducing colonic carcinogenesis [72]. Therefore, the trophic effects on colonocytes of folate-producing strains deserve to be evaluated. Moreover, the supply of folate by bifidobacteria may also contribute to lower hyperhomocysteinemia, since the administration of folate-producing *B. longum* exerted beneficial effects on the homocysteine levels of hemodialysis patients [73].

Besides their exploitation as an endogenous source of vitamin, folate-producing bifidobacteria may also be used to fortify fermented dairy products, as milk is a poor source of folate. This concept was tested in a particular study where seven strains of *Bifidobacterium* were evaluated for their capacity to enhance the folate concentration of reconstituted skim milk, resulting in a strain of *B. breve* being selected as the most promising [56]. Moreover, mixed culture fermentations of reconstituted skim milk were successfully carried out using folate-producing strains of *Bifidobacterium* in conjunction with strains of *Streptococcus thermophilus* and/or *Lactobacillus delbrueckii* subsp. *bulgaricus* from conventional yogurt, demonstrating that it is possible to increase folate levels in fermented milk products through appropriate selection of bacterial strains.

## 7. Conclusions

The use of folate-producing strains can be regarded as a new perspective on the specific uses of probiotics. Within the genus *Lactobacillus*, the strains belonging to the species *L. plantarum* are expected to produce folate in the presence of preformed pABA, while the other species cannot be regarded as folate producers. Therefore, the application of lactobacilli as folate-producing probiotics seems to be precluded, even though selected strains of *L. plantarum* deserve to be used in animal trials to provide evidence of their ability to produce folate *in vivo*. Unlike lactobacilli, several folate‑producing *Bifidobacterium* strains have been selected, but the release of high amounts of vitamin does not seem to be widespread within the genus. Animal trials confirmed that the administration of folate-producing bifidobacteria positively affected the plasmatic folate level, indicating that the vitamin is produced *in vivo* by the probiotic strains, and absorbed. In a human trial, the administration of the same strains resulted in a significant increase of folate concentration in feces. Even though the effect on plasmatic levels has not been investigated so far, folate-producing bifidobacteria may provide a complementary endogenous source of the vitamin and may contribute to prevent folate deficiency, which is often associated with premalignant changes in the colonic epithelia.
